# Chronic Responses of *Daphnia magna* Under Dietary Exposure to Leaves of a Transgenic (Event MON810) Bt–Maize Hybrid and its Conventional Near-Isoline

**DOI:** 10.1080/15287394.2015.1037877

**Published:** 2015-08-11

**Authors:** Daniel Ferreira Holderbaum, Marek Cuhra, Fern Wickson, Afonso Inácio Orth, Rubens Onofre Nodari, Thomas Bøhn

**Affiliations:** ^a^ Federal University of Santa Catarina, Graduate Program in Plant Genetic Resources, Florianópolis, Brazil; ^b^ GenØk, Centre for Biosafety, Tromsø, Norway; ^c^ UIT, The Arctic University of Norway, Faculty of Health Sciences, Tromsø, Norway

## Abstract

Insect resistance is the second most common trait globally in cultivated genetically modified (GM) plants. Resistance is usually obtained by introducing into the plant’s genome genes from the bacterium *Bacillus thuringiensis* (Bt) coding for insecticidal proteins (Cry proteins or toxins) that target insect pests. The aim of this study was to examine the hypothesis that a chronic, high-dose dietary exposure to leaves of a Bt–maize hybrid (GM event MON810, expressing a transgenic or recombinant Cry1Ab toxin), exerted no adverse effects on fitness parameters of the aquatic nontarget organism *Daphnia magna* (water flea) when compared to an identical control diet based on leaves of the non-GM near-isoline. Cry1Ab was immunologically detected and quantified in GM maize leaf material used for *Daphnia* feed. A 69-kD protein near Bt’s active core-toxin size and a 34-kD protein were identified. The *D. magna* bioassay showed a resource allocation to production of resting eggs and early fecundity in *D. magna* fed GM maize, with adverse effects for body size and fecundity later in life. This is the first study to examine GM-plant leaf material in the *D. magna* model, and provides of negative fitness effects of a MON810 maize hybrid in a nontarget model organism under chronic, high dietary exposure. Based upon these results, it is postulated that the observed transgenic proteins exert a nontarget effect in *D. magna* and/or unintended changes were produced in the maize genome/metabolome by the transformation process, producing a nutritional difference between GM-maize and non-GM near-isoline.

Through the use of recombinant DNA (rDNA) technology (genetic engineering), genes from the soil bacterium *Bacillus thuringiensis* (Bt) coding for insecticidal δ-endotoxins, known as Cry-proteins or Cry-toxins, have been transferred into important crop species, including maize (*Zea mays* L.) (Center for Environmental Risk Assessment [CERA], 2015). This genetic recombination enables economically important crop plants to produce their own insecticide, in order to protect them from insect pests. A recombinant Cry1Ab (henceforth named rCry1Ab) was the first Bt-protein used in commercial transgenic maize (YieldGard maize, GM event MON810, unique identifier MON-ØØ81Ø-6) (CERA, 2015).

The construct designed for insertion in the MON810 maize event contained the cauliflower mosaic-virus (CaMV) 35S promoter, the maize heat shock protein intron, the Bt *cry1Ab* gene for resistance to lepidopterans, and the nopaline synthase termination sequence, which was lost by truncation upon random insertion of expression cassette into maize’s genome (Rosati et al., 2008). By means of conventional breeding, the same transgene expression cassette present in MON810 was introgressed into several maize hybrids, many of which are still cultivated today (La Paz et al., 2014). Further, MON810 is also used in some of the so-called “stacked” GM events, which combine more than one single GM event by means of conventional breeding (Taverniers et al., 2008). Among hundreds of commercial GM events, event MON810 has the third highest number of regulatory approvals globally (James, 2014).

Bt spores and crystalline insecticidal toxins have been employed as a means of biological control in organic agriculture since the 1920s (Lemaux, 2008). However, use of Cry-toxins in GM plants involves some important differences. Firstly, Cry1Ab is expressed in *B. thuringiensis* only during sporulation, in crystalline inclusions of an inactive pro-toxin of 130 kD, which need to be cleaved under specific conditions such as high pH and presence of certain proteases and to find specific receptors in the gut of target organisms to become active and toxic (Bravo et al., 2007). In MON810 maize, the *cry1Ab* gene is truncated, and according to the manufacturer, codes for a pre-activated 91-kD truncated rCry1Ab toxin (CERA, 2015). The rCry1Ab toxin is expressed continuously but in different quantities in various tissues in transgenic maize throughout the life cycle of the plant (Székács *et* al., 2010a), creating a different exposure scenario for nontarget organisms than occurs through either pro-toxin expression by the bacterium itself or as used in pesticidal Bt sprays.

Despite global adoption of MON810 Bt–maize, there is scientific and public disagreement concerning the risks these plants may pose to the environment (Bøhn et al., 2012; Ricroch et al., 2010; Romeis et al., 2013; Wickson et al., 2013). Recently, Bt–maize and particularly MON810 maize were the focus of scientific controversy in the United States (U.S. Environmental Protection Agency [EPA], 2010, pp. 67–68; Waltz, 2009), while in Europe, despite a European approval for cultivation of MON810 maize, controversial temporary prohibitions on cultivation were implemented in Austria, France, Germany, Greece, Luxemburg, and Hungary, based upon what these countries noted as scientific evidence of risk of adverse effects to biodiversity (Bøhn et al., 2012; Wickson et al., 2013). Within both science and policy settings, evidential basis for evaluating the potential risk of adverse effects from MON810 maize to nontarget organisms and biodiversity remains a topic of ongoing debate (Bøhn et al., 2012; Ricroch et al., 2010; Romeis et al., 2013; Wickson et al., 2013).

Several studies reported no adverse effects of Bt maize on nontarget organisms, and pest control with Bt–plants seems to be less harmful than broad-spectrum chemical insecticides (Marvier et al., 2007; Naranjo, 2009). In contrast, negative nontarget effects of Bt–maize were found in certain cases, particularly for aquatic organisms, such as *Trichoptera* larvae fed Bt–maize leaves (Rosi-Marshall et al., 2007; Chambers et al., 2010), and *Daphnia magna* fed ground Bt–maize kernels (Bøhn et al., 2008, 2010). Significant effects of MON810 maize were recently noted in salmon (*Salmo salar*) (Gu et al. 2013, 2014), but were considered to be of minor biological significance because the overall development and health of the animals was not affected. Nontarget effects of other Bt–plants (*Populus* spp.) were also reported for aquatic biota (Axelsson et al., 2011a, 2011b).

*Daphnia magna* (water flea; Cladocera–Crustacea) (Figure 1) is a model organism widely advocated and employed in international standards and protocols for short-term (acute) and long-term (chronic) exposure studies for determining substance toxicity, as well as a more general model for studies of genomic, ecological and evolutionary responses (Colbourne et al., 2011). *Daphnia magna* feeds on suspended particles of organic material such as unicellular algae, bacteria, yeast, and particulate biomass such as detritus, and plays critical roles as both consumer and prey in lentic systems (Lampert, 2006). Under favorable environmental conditions, 1 to 2 wk after eclosion, *D. magna* produces diploid subitaneous eggs that are ready for immediate development of female offspring by means of parthenogenesis. However, under stressful conditions, individuals may start to (i) produce parthenogenic diploid males, allowing for subsequent sexual reproduction and/or (ii) deposit diapause eggs in protective structures (ephippia) (Figure 1). The diapause eggs may be of either sexual or asexual origin, depending upon environmental conditions (Vollmer, 1960; Bouchnak and Steinberg, 2010).

**FIGURE 1.**
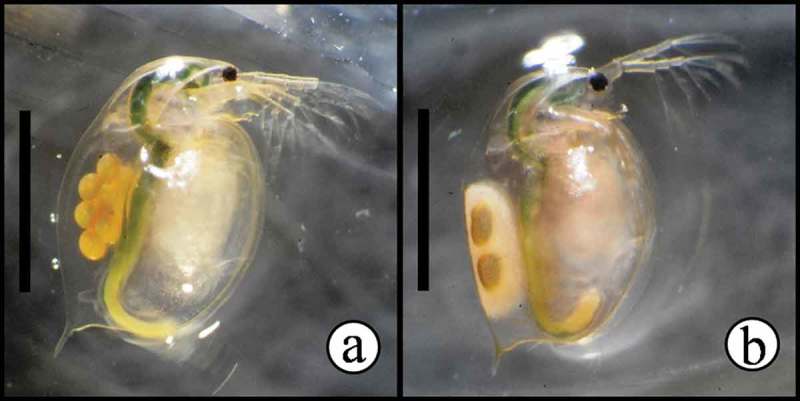
Examples of experimental *D. magna* fed maize leaves. Maize leaf-particles can be seen in the gut of animals. a) *D. magna* bearing subitaneous eggs. b) *D. magna* bearing an immature ephippium (protective structure enclosing two dormant eggs). Bars have 2 mm.

The aim of this study was to compare quality of a genetically modified (GM) maize (MON810) hybrid and its appropriate non-GM counterpart as feed for *D. magna* in a 42-d bioassay with chronic, high-dose dietary exposure to plant leaf material. In the case of maize, near-isogenic lines are widely considered the appropriate comparators, as they share the same genetic constitution except for the target gene/sequence, in this case, the MON810 *cry1Ab* gene expression cassette, and possibly other genes with genetic linkage to the target sequence (Zeven and Waninge, 1986). MON810 Bt–maize leaves are known to contain 5- to 20-fold higher Bt-toxin levels than maize kernels (Nguyen and Jehle, 2007), and to our knowledge, Bt-transgenic plant leaves have not previously been tested with the *D. magna* model. The hypothesis examined in this study was compared to its non-GM near-isogenic line (PAN 6Q-121), GM-maize hybrid PAN 6Q-321B (event MON810) does not exert any adverse effects on fitness parameters of *D. magna* during a full-life-cycle feeding experiment under chronic, high-dose dietary exposure to maize-leaf feed.

## MATERIALS AND METHODS

### Plant Material

Maize plants from the GM hybrid PAN 6Q-321B (event MON810) and from its non-GM near-isogenic line PAN 6Q-121 (white maize varieties cultivated in South Africa) were cultivated in growth chambers under equal conditions of soil type, nutrient supply, photoperiod, and luminosity, and varying temperature and soil-humidity regimes. The leaves from six random maize plants (three GM plants and three non-GM plants) at the beginning of R3 developmental stage (milky grain) were collected, weighed, lyophilized, and stored at room temperature until use.

### Maize Leaf Sample Processing and Feed Preparation

Samples taken from the middle longitudinal portion of the lyophilized maize leaves (Székács et al., 2010b), excluding the central rib, were ground in a FastPrep grinder (MP Biomedicals, Illkirch, France) until attainment of a fine powder, and then stored at −70°C until used for *D. magna* feed preparation. In order to ensure the provision of feed with particles of edible size for *D. magna* (≤50 μm), 700 mg, a quantity previously established in preliminary tests, of leaf powder from each of the 6 plants was individually mixed with 200 ml ultrapure water and the resulting feed solutions were filtered in previously weighed plankton nettings (mesh size 50 μm). Filtered feed solutions were kept at 2°C, while the filtrates in the plankton nettings were dried in an oven at 40°C until attainment of constant weight (approximately 48 h). The amount of retained material in the plankton nettings was weighed and the quantity of material in the feed solutions (particle size ≤50 μm) was calculated by the difference. Feed solutions were then diluted to 1 mg organic carbon/ml (mg C/ml)—considering 41.27% of carbon in dry maize leaves (Latshaw, 1924) —and were aliquoted and stored at −70°C until fed to *D. magna*, with one aliquot thawed per day per feed solution to feed the experimental animals.

### Maize Leaf Total Protein Determination

Total protein was extracted with the P-PER Plant Protein Extraction Kit (Pierce Chemical Co., Rockford, IL) following the manufacturer’s recommendations, with addition of cOmplete protease inhibitor cocktail (1X) (Roche, Mannheim, Germany) to the P-PER kit working solution, at 1:100 proportion. Total protein concentration was determined by the Bradford (1976) method, using bovine serum albumin (BSA) as a standard (more detailed information in Electronic Supplementary Material 1 [file ESM1.pdf]).

### Identification of rCry1Ab

Maize leaf total protein extract samples were prepared for sodium dodecyl sulfate (SDS) polyacrylamide gel electrophoresis (PAGE) and analyzed by immunoblotting. Purified bacterial Cry1Ab purified from recombinant *Escherichia coli* (Abraxis, Warminster, PA) was used as a positive control. Blots were detected through binding with polyclonal rabbit-anti-Bt Cry1Ab antibody (Abraxis, Warminster, PA) (primary antibody), and goat anti-rabbit immunoglobulin (Ig) G antibody (secondary antibody) conjugated to alkaline phosphatase (AP) (Pierce Chemical Co., Rockford, IL). Protein mass was estimated with a calibration curve based on the natural logarithm of molecular weights of MagicMark XP Western Protein Standards (Life Technologies Corporation, Paisley, UK) and their relative mobility in SDS-PAGE, using the migration distance of the lowest molecular weight standard protein (20 kD) as the relative mobility marker (more detailed information in Electronic Supplementary Material 1 [file ESM1.pdf]).

### Quantification of rCry1Ab in Maize Leaf Feed

rCry1Ab was quantified in maize leaf feed by means of Agdia Cry1Ab/Cry1Ac DAS enzyme-linked immunosorbent assay (ELISA) kit (Double Antibody Sandwich ELISA) (Agdia, Elkhart, IN) following the manufacturer’s recommendations, with adaptations needed for the quantification of Cry1Ab as follows: A standard curve of serial dilutions of bacterial Cry1Ab (Abraxis, Warminster, PA) was constructed and used to estimate MON810 rCry1Ab content in maize leaf feed solutions (more detailed information in Electronic Supplementary Material 1 [file ESM1.pdf]).

### Rearing of Daphnia magna Populations

The *D. magna* clonal population was reared for several generations prior to the experiment in M7 medium (Elendt, 1990; Organization for Economic Cooperation and Development [OECD], 2008), fed *Scenedesmus dimorphus* green algae. *Scenedesmus dimorphus* was cultivated in growth medium for unicellular green algae (Kuhl and Lorenzen, 1964) in 1-L round glass flasks at 23 ± 2°C, under continuous aeration and artificial light from full-spectrum fluorescent lamps (75 uE/m^2^/s).

### Experimental Design and Setup of Daphnia magna Bioassay

*Daphnia magna* born within 24 h, from the second clutch of females of a single clone (courtesy of Dag Hessen, Department of Biology, University of Oslo, Oslo, Norway), were used for the 42-d *D. magna* bioassay. Animals were randomly assigned to two diets: feed prepared from leaves of Bt–maize (GM) or feed prepared from near-isogenic maize (NM). The experiment was conducted as a completely randomized mixed factorial design, with fixed factors *maize variety* (Bt–maize [GM] and the non-GM near-isogenic line [NM]) and *time* (animal age in days), and a random factor *plant* (six random plants). Two hundred and ten animals were randomly allocated to numbered 100-ml beakers filled with M7 medium, with one animal per beaker. Ninety of these were fed leaves of the GM maize and another 90 animals were fed leaves of NM maize, with 30 animals receiving feed from each individual plant used in each group. Thirty additional animals were fed green algae *S. dimorphus*, in order to verify whether requisites for the test’s validity were met according to OECD guidelines for *Daphnia* reproduction testing (OECD, 2008), where at least 10 animals are recommended for the control group. Test animals were fed 0.2 mg C of leaf powder (200 μl maize leaf solution) or 0.2 mg C of *S. dimorphus* cells daily. The temperature and light regime of the *Daphnia* culture and experiment was as follows: temperature range: 14.2–23.6°C; average minimum: 19.9 ± 1.4°C; average maximum: 21.7 ± 0.7°C; light: 24 h continuous artificial light from fluorescent lamps. The experiment was set up and conducted as a blind study: A color code was assigned for the leaf-particle solutions by a third party not involved in the experiment, and the code for feeding treatments was only revealed after the experiment and all measurements were completed.

### Data Collection for Daphnia magna Bioassay

Each experimental animal (the experimental unit) was inspected daily for survival. Animals without a heartbeat visually inspected under a stereomicroscope for more than 10 s were considered dead. Every individual was confirmed to be female by visual inspection of sexual dimorphism. The incidence of reproduction and time to first reproduction (both observed at the first clutch) were determined by daily inspection, and production of ephippia (resting eggs—protective, saddle-shaped structures that enclose diapause [dormant] eggs; Figure 1) was also recorded daily. For practical reasons and to diminish *D. magna* stress, fecundity and size were recorded on predefined days. Fecundity (number of live neonates) was counted every 3 d, after transferring the parent animals to new beakers containing fresh medium, filtering the offspring in plankton netting, and counting them under the stereomicroscope. All experimental animals and a 2-mm scale (precision of 0.01 mm) were photographed at d 9, 18, 27, 36, and 42 with a digital camera attached to a stereomicroscope, and body size (the length from the top of the head to the base of the caudal spine) was measured using the ImageJ software (Rasband, 1997–2008).

### Data Analysis

Analyses were carried out within a Generalized Linear Mixed Model (GLMM) framework, unless otherwise stated. Considering plants were collected at random from a population of GM and NM maize plants, *plant* was included as a random-effect variable in the models for all endpoints, in order to account for the potential variance between individual plants. The inclusion of the variability from random effects in statistical models improves standard errors estimation, allowing for more robust inferences, and further extending these inferences to the population from which the random samples (the random levels of *plant*, in this case) were collected, instead of limiting inferences to the specific levels of the random variable (Bolker et al., 2009). Conditions for the reliable estimation of random effects include having >5–6 random effect levels per random effect and >10–20 samples per treatment levels (Bolker et al., 2009), both conditions satisfied in this study.

*Daphnia magna* survival and time to first reproduction were analyzed in frailty models, which extend the Cox proportional hazards model to accommodate random effects (Hougaard, 2000). The incidence of reproduction and production of ephippia among *D. magna* was analyzed in binomial (logistic) models. Repeated-measures data from body size (continuous variable, normal distribution) and stage/cumulative fecundity (count variables, Poisson distribution) were analyzed in mixed and generalized mixed regression models, respectively, including as fixed effects *maize variety, time* (regarded as a continuous variable), and interaction of *maize variety* and *time*, plus quadratic and cubic terms for *time* when appropriate, according to previous exploratory analysis and model selection criteria. When applicable, the dispersion parameter (generalized chi-squared/degrees of freedom) and the Bayesian information criterion (Schwarz, 1978) were used for assessment of model fit and model selection.

Effects, parameters and associations were considered significant when *p* < .05. Confidence intervals (CI) (95%) of relevant statistics are reported. Model selection statistics are compiled in Electronic Supplementary Material 2 (file ESM2.pdf), and inferential statistics for all end-points are compiled in Electronic Supplementary Material 3 (file ESM3.pdf). Analyses were carried out in SAS 9.3 (SAS Institute, Inc., Cary, NC), using the procedures GLIMMIX, MIXED, and PHREG for GLMMs, LMMs, and frailty models/Cox regression, respectively (more detailed information in Electronic Supplementary Material 1 [file ESM1.pdf]). Raw data for all endpoints are provided in Electronic Supplementary Material 4–10 (files ESM4.pdf, ESM5.pdf, ESM6.pdf, ESFM.pdf, ESFM.pdf, ESM9.pdf, ESM10.pdf).

## RESULTS

### Identification and Quantification of rCry1Ab

**FIGURE 2.**
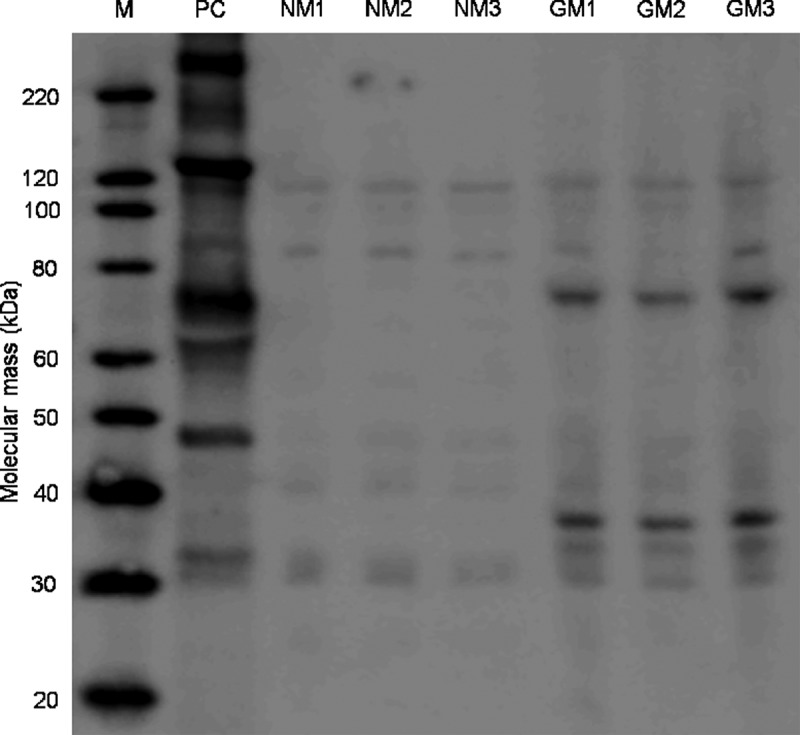
Immuno-detection of MON810’s rCry1Ab. MagicMark™ XP Western Protein Standard (M), positive control (PC - Cry1Ab purified from B. thuringiensis), the three non-transgenic near-isoline plants (NM1, NM2 and NM3) and the three MON810 Bt-maize plants (GM1, GM2 and GM3). Strong immunoreactive bands of ca. 69 kDa and 34 KDa can be distinguished in the GM-maize samples.

rCry1Ab was identified in maize leaf total protein extracts by immunoblotting, only in protein extracts of leaves from the three GM-maize plants (Figure 2). Two strong Cry1Ab immunoreactive bands were present in GM (MON810) maize samples, in all immunoblot membranes. One band was estimated with a mass of approximately 69 kD (close to the 65-kD active core toxin of *Bt*) and the other of approximately 34 kD. Similarly, rCry1Ab was detected by ELISA only in the three GM-maize feed solutions, and estimated at 2530, 1880, and 2040 ng/g leaf dry mass for each feed solution (GM1, GM2, and GM3, respectively). Thus, *D. magna* fed Bt–maize leaves in this experiment were given doses of 1.22, 0.91, and 0.99 ng rCry1Ab/d (0.484 mg of maize leaf-particles/d). If the rCry1Ab in the leaf solutions was completely stable and dissolved in the *D. magna* medium (100 ml), and considering that each animal received 3 consecutive doses before each medium renewal, the maximum possible concentration of rCry1Ab in the daphnia medium throughout the experiment would be 36.6, 27.3, and 29.9 ng/L, in GM1, GM2, and GM3 feed solutions, respectively.

### Daphnia magna Chronic Bioassay

*Daphnia magna* fed algae showed 20% mortality at d 21, by which time their mean cumulative fecundity was 114 juveniles, conforming to international guidelines (OECD, 2008). *Daphnia magna* fed algae started to reproduce at d 8 and did not produce any ephippia. *Daphnia magna* fed maize-leaf diets displayed higher rate of mortality and produced less offspring and ephippia, indicating suboptimal feed conditions. However, *D. magna* fed maize leaves did reproduce regularly and upheld viable populations that might be compared across the GM-maize and NM-maize diets.

### Survival

Survival of *D. magna* provided with GM and NM maize diets did not differ significantly. The median survival time (the time at which 50% of the animals were alive) was 29 d and 26 d for GM and NM maize, respectively, and 95% CI included means throughout the experimental period.

### Body Size

**FIGURE 3.**
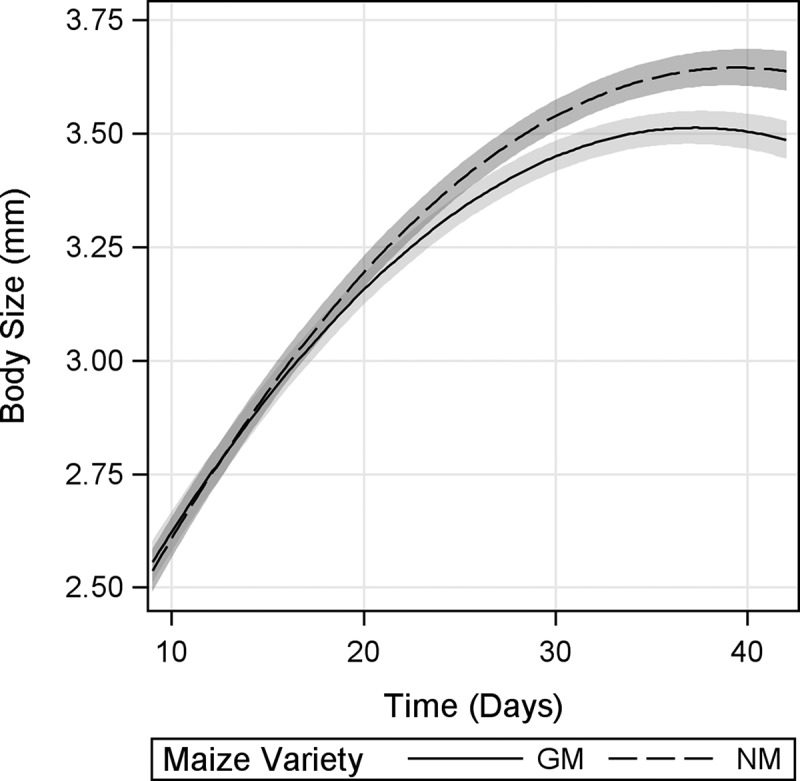
Body size of *D. magna* fed Bt-maize (GM) and near-isogenic maize (NM) leaves. Shaded bands indicate 95% confidence intervals.

For body size, a significant *maize variety* × *time* interaction was found, indicating different growth trends between *D. magna* under GM and NM maize diets (Figure 3). From d 9 to approximately d 24, *D. magna* body size was not markedly influenced by diet. However, from approximately d 27 onward, *D. magna* fed GM-maize leaves displayed a significantly smaller body size than those fed NM leaves, as evidenced by nonoverlapping 95% CI. The size difference between GM and NM maize-fed *D. magna* reached about 4.5% by the end of the experiment.

### Incidence of Reproduction

No significant association was established between diet and incidence of reproduction in *D. magna*. In total, 151 animals (of 180 fed maize leaves) had at least one reproductive event, 77 fed GM-maize leaves and 74 fed NM-maize leaves.

### Age at First Reproduction

No significant association between diet and age at first reproduction was established. Median age at first reproduction was 11 and 12 d for animals fed GM and NM maize diets, respectively.

### Stage Fecundity

**FIGURE 4.**
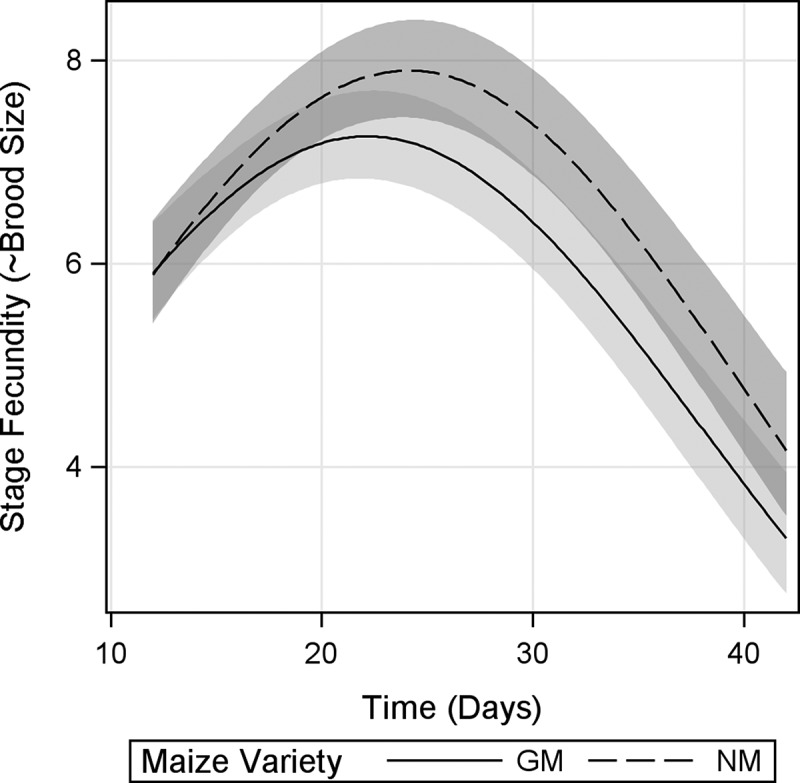
Stage fecundity of *D. magna* fed Bt-maize (GM) and near-isogenic maize (NM) leaves. Shaded bands indicate 95% confidence intervals.

Stage fecundity—an approximation of clutch size obtained by counting the number of juveniles every 3 d—showed a significant *maize variety* × *time* interaction, indicating different reproduction trends of *D. magna* under GM-maize and NM-maize diets (Figure 4). Inspection of CI revealed significant differences from d 24 until d 39, with *D. magna* fed GM-maize diet giving birth to on average 1 juvenile less than *D. magna* fed NM-maize per stage (every 3 d). Further, regression curves showed that *D. magna* fed GM-maize diet reached its maximal stage fecundity (peak of reproductive effort) before *D. magna* fed NM-maize diet (Figure 4), indicating a resource allocation for earlier fecundity.

### Cumulative Fecundity

**FIGURE 5.**
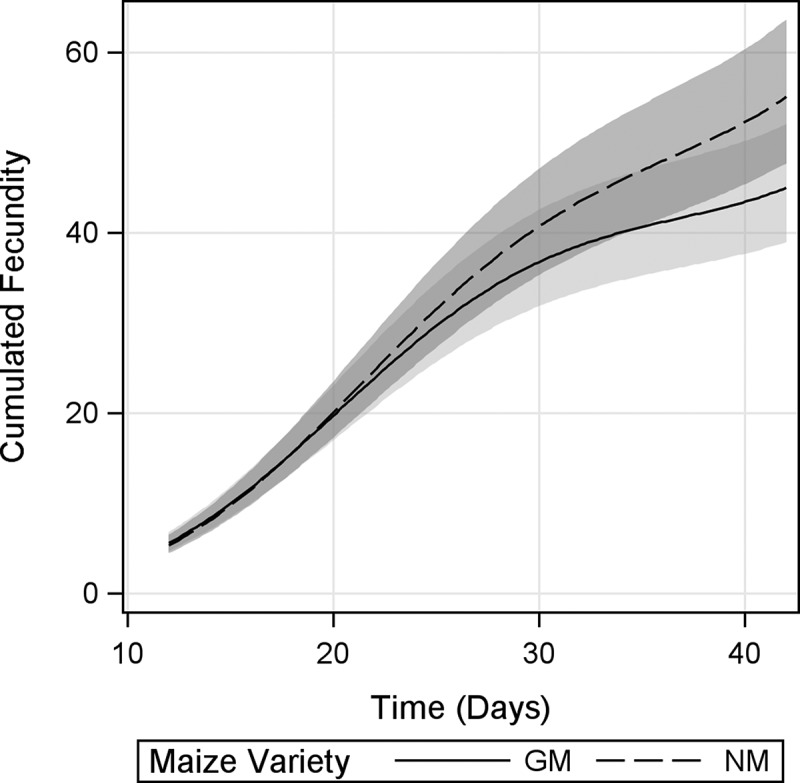
Cumulative fecundity of *D. magna* fed Bt-maize (GM) and near-isogenic maize (NM) leaves. Shaded bands indicate 95% confidence intervals.

For cumulative fecundity a significant *maize variety* × *time* interaction was evidenced, indicating that the mean cumulative fecundity rates (the slopes of the regressions) were different between groups (Figure 5). However, inspection of 95% CI showed no significant differences in cumulative fecundity at any specific time point. The difference between cumulative fecundity rates and total fecundity of *D. magna* under both diets demonstrated a tendency to get larger with time (hence the significant interaction), with *D. magna* fed GM maize reproducing approximately 20% less offspring as a consequence of the different reproductive rates by d 42.

### Ephippia Production

**FIGURE 6.**
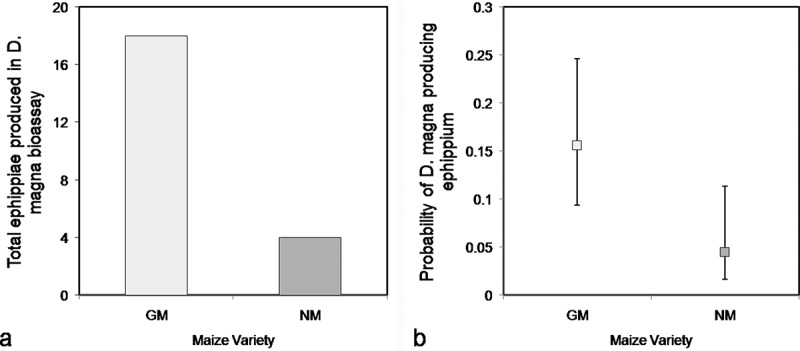
Ephipum production in *D. magna*. a) Total ephippia produced in the Daphnia bioassay, for experimental *D. magna* fed Bt-maize (GM) and near-isogenic maize (NM) leaves. b) Probability of ephippium production among experimental *D. magna* fed Bt-maize (GM) and near-isogenic maize (NM) leaves. Error bars indicate 95% confidence intervals.

A significant association was found between ephippia production and the diet provided to *D. magna*. In total, 18 daphnids (10%) produced ephippia during the experiment; 14 of these were fed GM maize and 4 were fed the NM maize. Four daphnids fed GM-maize leaves produced an ephippium twice, so that in total 22 ephippia were produced during the experiment, by animals under both diets combined (18 ephippia in the GM diet and 4 ephippia in the NM diet) (Figure 6a). The binomial model only considered whether animals produced or did not produce ephippia (a dichotomous response). Animals fed GM maize produced significantly more ephippia, with a 3.5 fold greater probability of producing an ephippium than animals fed NM maize (odds ratio [OR] = 3.96, 95% CI: 1.24, 12.65; risk ratio [RR] = 3.5, 95% CI: 1.23, 8.36) (Figure 6b).

## DISCUSSION

This study demonstrated chronic effects of a GM Bt-maize hybrid (PAN 6Q-321B, event MON810) on aquatic non-target organism *D. magna* fed maize leaves. Significant differences include an increased ephippia production - a response indicative of stressful conditions - and reductions in body size, stage- and cumulative- fecundity rates at middle to later life-stages for animals fed GM-maize. On the other hand, no differences were found between maize varieties for incidence of reproduction, age at first reproduction, and survival.

Ephippia production increased by 3.5-fold in *D. magna* fed GM maize, indicating an important biological effect. Under stressful environmental conditions such as drought, pollutant accumulation, and food scarcity, production of resting eggs (or ephippia) may be advantageous for *Daphnia* females (Ban et al., 2009). This may be attributed to resting eggs presenting a high chance of enduring adverse conditions and hatching when the environment becomes suitable again. Usually cladocerans produce male offspring during the emergence of detrimental conditions and subsequently produce sexual eggs and ephippia after mating, although asexual production of ephippial eggs is also possible (Hebert, 1978). Ephippia observed in our study may have been produced asexually since all experimental animals were identified as female, isolated from each other, and juveniles remained with their mother for a maximum of 3 d.

Two other studies found ephippia containing diapause eggs without previous sexual fertilization in *D. magna* (Vollmer, 1960; Bouchnak and Steinberg, 2010), both under conditions of low nutritional quality. Considering that the animals fed green algae did not produce epphipia, while the animals under GM and NM maize leaf diets did produce them, one might speculate that the maize leaf diets were nutritionally inferior compared to green algae. However, the higher ephippia production in *D. magna* fed GM maize also suggests a significant qualitative difference between the GM-maize hybrid and its NM near-isogenic counterpart, that may be not only in terms of the expression of Bt-toxin but also potentially in their nutritional quality. Further, given that ephippia production was a rare event in these study conditions, even larger sample sizes (number of test animals under different treatments) need to be used in order to achieve greater certainty for estimations.

The reductions in growth rate and stage fecundity in *D. magna* fed GM-maize occurred after the period of maximum reproductive output (between d 21 and 24; Figures 3 and 4, respectively), and the observed slower rate of increase in cumulative fecundity (Figure 5) likely reflects reduced stage fecundity rate. In addition, *D. magna* fed GM-maize leaves reached reproductive peak earlier, suggesting a resource allocation to early fecundity. Although the effects on fecundity rates were small, dependence of the influence of *maize variety* (GM maize vs. NM maize) on *time* indicated that negative effects of GM maize mostly appeared in late life stages, resulting in a 20% lower mean total fecundity at the end of the experiment. This is no trivial reduction and needs to be considered as an important biological effect. In order to explain these results, one needs to bear in mind that *D. magna* growth and fecundity are known to be affected by both adverse effects (Villarroel et al., 2003; Heugens et al., 2006), and food quality (Heugent et al., 2006; Müller-Navarra, 1995; Stige et al., 2004).

Depending on the toxicity level, the concentration of the toxic substance, and other environmental conditions, adverse effects on *D. magna* may produce different life-history configurations. This includes (i) delayed reproduction and reduced clutch size and total fecundity at low toxin concentrations, with additional negative effects on body size and survival at high toxin concentrations (Villarroel et al., 2003; Heugens et al., 2006); (ii) severely impaired growth, reproduction, and survival (high toxicity); and (iii) early-life fecundity compensation at the expense of later growth and survival (low toxicity) (Bøhn et al., 2008).

A lower food quality may also exert on *D. magna* various effect scenarios, such as reduced growth rate and delayed onset of reproduction (Stige et al., 2004), decreased growth rate and smaller clutch size (Müller-Navarra, 1995), and diminished fecundity and increased ephippia production (Bouchnak and Steinberg, 2010). Further, low food quality and low toxicity may act together on *D. magna* fitness, producing malnutrition and impairment of feeding and digestion, respectively, resulting in less energy available for maintenance, growth, and reproduction (Dao et al., 2010).

The results obtained for growth and fecundity in this experiment suggest a response to stressful conditions of *D. magna* fed GM maize, that is, a resource allocation for ephippia production and early reproductive effort, with reduced growth and fecundity later in life. The observed life-history configuration of *D. magna* fed GM maize leaves, compared to NM maize leaves, leads us to hypothesize that a lower feed quality of the GM maize leaves and/or a toxic effect of the rCry1Ab are the causal factors, because both conditions, separately or together, might create such a life-history scenario. What can be inferred with greater certainty, however, is that a potent adverse effect is likely absent since there was no apparent indication of a lethal effect in *D. magna* fed GM maize leaves.

In contrast with these results, Bøhn et al. (2008) observed an earlier onset of reproduction and reduced survival in *D. magna* fed MON810 Bt–maize kernels of a different hybrid, suggesting a weak adverse effect of rCry1Ab, for which the concentration was approximately 30-fold lower than in leaf feed used in this study. This apparent discrepancy seems to indicate the absence of a toxic effect of the MON810 rCry1Ab in *D. magna*, but one needs to bear in mind that the influence of rCry1Ab (or any other toxin) might be modulated by food quantity and quality (Heugens et al., 2006; Chandini, 1989).

Based on a range of fitness parameters and on knowledge of life-history theory, the rDNA technology employed in the development of GM plants, and specific recombinant trait and GM event under study, possible mechanisms behind the observed effects in *D. magna* fitness may be postulated. Overall, differential effects observed for *D. magna* growth, fecundity, and ephippia production refute the hypothesis that a diet of leaves of this Bt–maize hybrid (event MON810) does not negatively impact the model organism’s fitness parameters under controlled, high-exposure conditions. On the other hand, no significant effects were observed on the incidence of reproduction, time to first reproduction, and survival of *D. magna* fed GM maize leaves. Based on these results, two nonexcluding causal mechanisms related to transgenic event MON810’s characteristics are proposed and discussed:

1. The random insertion of specific recombinant DNA sequences into maize’s genome (producing an intentional effect) may unintentionally cause other changes (i.e., pleiotropic or epigenetic effects), which might lead to formation of new metabolites or alter expression levels of existing metabolites in GM maize, either through silencing or up-/downregulation of specific genes, as demonstrated by others (Zolla et al., 2008; Agapito-Tenfen et al., 2013; La Paz et al., 2014). Further, the specific metabolic changes may depend on the specific GM event and the genetic background in which the recombinant locus was introgressed (i.e., the different hybrids) (La Paz et al., 2014).
2. The truncated, readily active rCry1Ab toxin expressed in MON810 maize and/or the detected rCry1Ab-related proteins/fragments (the existence of which in fact constitutes unintended effects) might exert a low toxic effect in *D. magna* (Bøhn et al., 2008), through an as yet unknown mode of action. In addition, inside MON810 maize, rCry1Ab may undergo posttranslational modifications that otherwise would not happen in bacteria, and thus also influence toxicity (Franck-Oberaspach and Keller, 1997).

Two strong immunoreactive signals of proteins with estimated mass of 69 and 34 kD were detected in the Bt–maize plants. Other investigators also found immunoreactive rCry1Ab proteins (or protein fragments) of several sizes in MON810 Bt–maize, including a 34-kD protein (Paul et al., 2010; Grubber et al., 2011). Further, the 34-kD protein apparently has greater stability to proteolysis, which might be relevant for potential effects of GM maize in agroecosystems (Paul et al., 2010). To our knowledge, these peptides have not yet been specifically tested for toxicity to nontarget organisms.

Cry1Ab purified from bacteria, which was used as positive control in the immune-detection of MON810’s rCry1Ab, showed immunoreactive bands of several molecular masses, including the 130-kD band for the original Cry1Ab pro-toxin, a possible dimer of the pro-toxin (260 kD), and a 65-kD band for the active core-toxin, but not the 34-kD band as found in the Bt–maize extracts, indicating a distinguishable difference between the Cry1Ab expressed in bacteria and that expressed in the leaves of MON810 maize. In this regard, it should be noted that unidentified characteristics of recombinant Bt-toxins in Bt–plants might affect binding and host specificity (Crickmore, 2005), while toxicity of recombinant Cry toxins may also be influenced by synergisms with endogenous plant metabolites or other extrinsic factors (Then, 2010). Further, the specificity of Bt-toxins is known to be largely determined by subtle differences in proteolytic processing (Haider et al., 1986), both toxicity and expression may be affected by single amino-acid changes in the polypeptide chain (Ward et al., 1988; Alzate et al., 2010), and differences in posttranslational modification mechanisms between prokaryotic and eukaryotic biological systems may influence the toxic potential of transgenic proteins (Franck-Oberaspach and Keller, 1997).

Considering that MON810-expressed transgenic Cry1Ab protein shows unique ontogenetic characteristics and that this and other studies found adverse effects of MON810 maize in nontarget organisms, the risk assessment of these GM Bt–plants needs to include studies that evaluate biological effects in model organisms and use representative plant material and plant-extracted recombinant proteins for safety testing, instead of using microbial proteins that are judged sufficiently similar to plant recombinant proteins given a limited set of biochemical and functional criteria (Raybould et al., 2012). Therefore, enhanced effort is required to purify plant-produced recombinant proteins in preparative scale, and to employ them in bioassays that demand plant-produced native-state proteins for a more precise risk assessment of GM-plants.

In order to supplement knowledge generated in this study, and to answer pending questions regarding risks of GM MON810 maize, further research is necessary to establish (a) the extent to which the interaction between genetic background (different maize hybrids) and transgenic event (i.e., MON810 and others) affects results of feeding bioassays with nontarget organisms using full-life-cycle studies, and (b) the molecular mechanisms underlying possible effects of MON810 Bt–maize on nontarget organisms.

## CONCLUSIONS

This study explored chronic effects of high dietary exposure of an aquatic nontarget organism to leaves of a MON810 GM Bt–maize hybrid, in comparison to its non-GM near-isoline. The GM Bt–maize diet resulted in increased ephippia (resting eggs) production, along with reductions in growth and fecundity later in the life cycle of our test organism, *D. magna*, indicating increased stress levels in animals fed GM Bt–maize leaves. It is postulated that the transgenic protein and/or unintended transformation-related changes in the expressed maize genome/metabolome were the causes for observed effects. Further studies are necessary to more precisely determine the mechanism or mechanisms underlying the sublethal fitness effects observed in *D. magna* exposed to Bt–maize leaves. Data indicate that effects of GM feed and food materials intended for lifelong consumption need to be tested on multiple endpoints, preferably over the full life cycle of model organisms, considering that important biological effects may only be detected after chronic exposure. Further, unique characteristics detected in the recombinant Cry1Ab of MON810 Bt–maize, as opposed to bacterially produced Cry1Ab, raise serious questions concerning whether microbial produced recombinant proteins are adequate for assessing risks posed by recombinant plant proteins. This highlights the importance of conducting safety testing for GM plants with plant material and plant-produced recombinant proteins, coupled to a case-by-case approach to GM-plant toxicity testing, in order to achieve more reliable accuracy in effect size and uncertainty estimation, and to contribute to the establishment of a more precise risk profile for specific GM plants/events.

## SUPPLEMENTAL DATA

Supplemental data for this article can be accessed at http://dx.doi.org/10.1080/15287394.2015.1037877

## FUNDING

This study was supported by a grant from the Norwegian Research Council under the MILJØ2015 program (project number 184107). DFH thanks Fredskorpset for financial support and preparation for a cultural/scientific exchange in Norway, CAPES for a master’s scholarship, and several colleagues at GenØk/UiT and UFSC for their support in this study. FW thanks the Norwegian Research Council for support to participate in this study through grant number 203288/S10.
